# Supplementary material for: The Influence of Filler Size and Crosslinking Degree of Polymers on Mullins Effect in Filled NR/BR Composites
Source: Polymers (Basel). 2021 Jul 12;13(14):2284. doi: 10.3390/polym13142284 (PMC8309370; doi:10.3390/polym13142284)
Supplement: Supplementary file 1 [file polymers-13-02284-s001.zip › polymers-1291455-supplementary.pdf]

polymers

MDPI

Supplementary Information

The influence of filler size and crosslinking degree of polymers on Mullins effect in filled NR/BR composites

Miaomiao Qian [1], Bo Zou [1], Zhixiao Chen [2], Weimin Huang [1], Xiaofeng Wang [2], Bin Tang [3], Qingtao Liu [4,*], Yanchao Zhu [1,*]

[1] College of Chemistry, Jilin University, Changchun 130012, China; qianmm19@mails.jlu.edu.cn (M.Q.); dr.z@jlu.edu.cn (B.Z.); huangwm@jlu.edu.cn (W.H.)
[2] State Key Laboratory of Inorganic Synthesis & Preparative Chemistry, College of Chemistry, Jilin University, Changchun 130012, China; zhixiaocheng18@mails.jlu.edu.cn (Z.C.); wangxf103@jlu.edu.cn (X.W.)
[3] Institute for Frontier Materials, Deakin University, Melbourne/Geelong, VIC 3216, Australia; bin.tang@deakin.edu.au
[4] Monash Institute of Pharmaceutical Sciences, Monash University, Melbourne, VIC 3800, Australia
* Correspondence: jason.liu@monash.edu (Q.L.); Yanchao_zhu@jlu.edu.cn (Y.Z.)

Milling procedure

Filler with different particle sizes was obtained via ball milling at different rotational speed. The detailed sample information is listed in Table 1.

Table S1. Various milling parameters in designed experiments.

Samples	Milling media	Rotational speed
M_1SiCB	None	400
M_2SiCB	None	600

Table S2. Mechanical properties of the rubber materials.

	Tensile strength (MPa)	Stress at 300% strain (MPa)	Elongation at break (%)
NR/BR	3.57	0.80	679.43

Preparation of MA grafted natural rubber (MNR_x)

The MNR_x was prepared using a laboratory-sized internal mixer (RM-200C, Harbin Hapro Electric Technology Co., Ltd., Harbin, China). Maleic anhydride (MA) and dicumyl peroxide (DCP) (10 wt% of MA) were mixed with NR in an internal mixer at 100 °C for 10 min. MA is used to functionalize the rubber matrix, and DCP is used to initiate the polymerization process. The interaction between NR and MA was further verified by FTIR spectra as shown in Figure S1. The new peaks in MNR at 1850 cm^{-1}, 1780 cm^{-1}, 1720 cm^{-1} and 1631 cm^{-1} were observed. These bands can be assigned to the grafted anhydride, which are due to symmetric and asymmetric C=O stretching vibration of succinic anhydride rings grafted on the NR molecule, respectively [1]. Therefore, the formation of graft copolymer of NR and MA was confirmed.

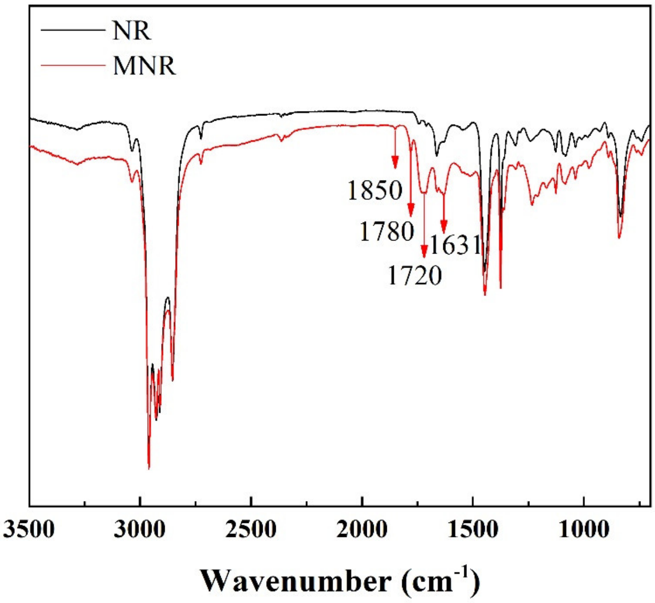

Figure S1. Infrared spectra of NR compared with MNR.

Uniaxial cyclic tension test

In order to illustrate the influence of the filler size and crosslinking degree on Mullins effect, cyclic uniaxial tension tests were performed on three different filled NR/BR composites. Tests were run at a constant strain rate of 0.025 s^{-1}. The samples were subjected to a cyclic uniaxial tension test with the maximum stretching increasing every 2 cycles. The stress-strain responses of different samples are shown in Fig.S2-8. It was observed that the applied stress needed for the samples to reach a given level of strain decreased after the first loading cycles, which was also termed as softening or the Mullins effect. The softening increased progressively with increasing maximum strain, and the degree of softening varied with the sample types even under the same strain. After the first few cycles, the samples responded coincide as increasing the test cycles.

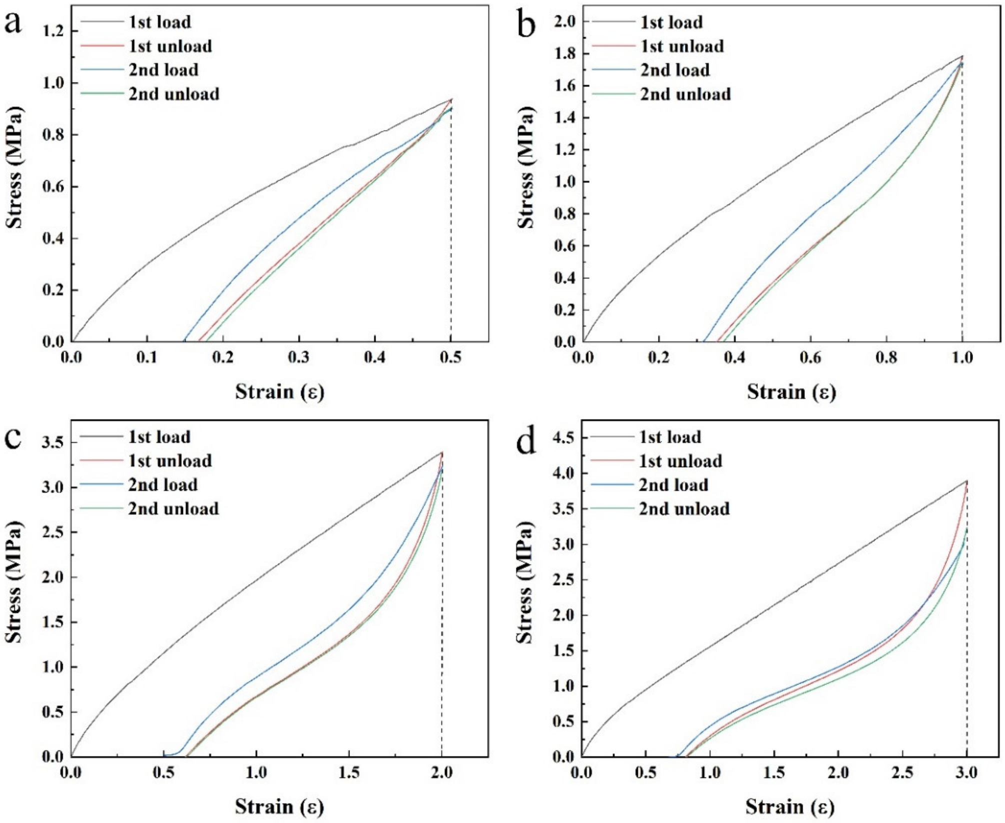

Figure S2. Stress-strain response of SiCB-NR/BR subjected to cyclic uniaxial tension: (**a**) ε = 0.5, (**b**) ε = 1.0, (**c**) ε = 2.0, (**d**) ε = 3.0.

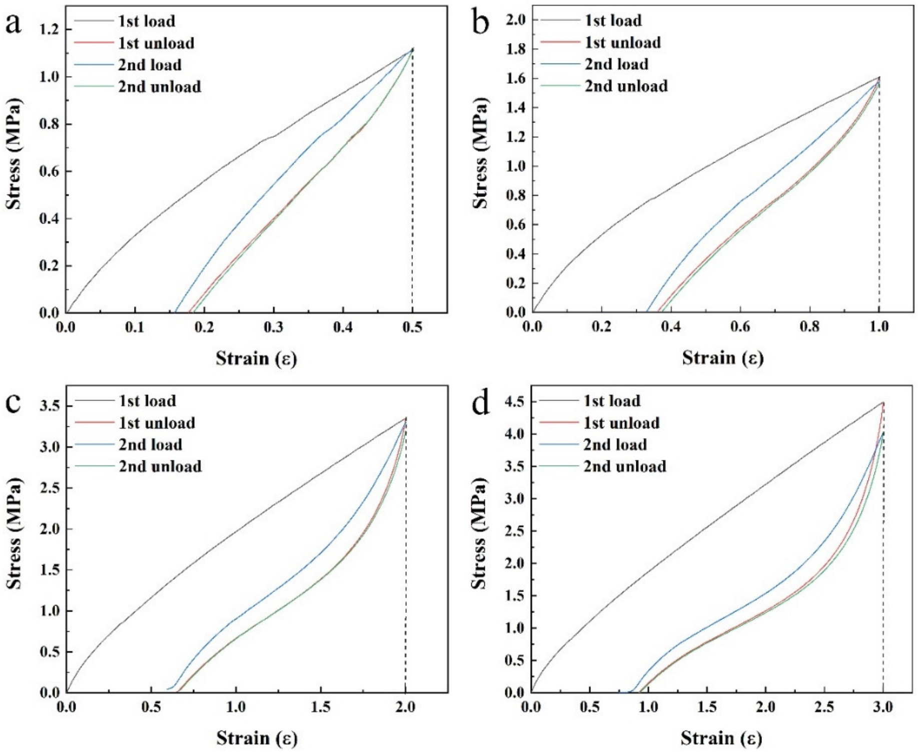

Figure S3. Stress-strain response of SiCB-MNR$_1$/BR subjected to cyclic uniaxial tension: (**a**) ε = 0.5, (**b**) ε = 1.0, (**c**) ε = 2.0, (**d**) ε = 3.0.

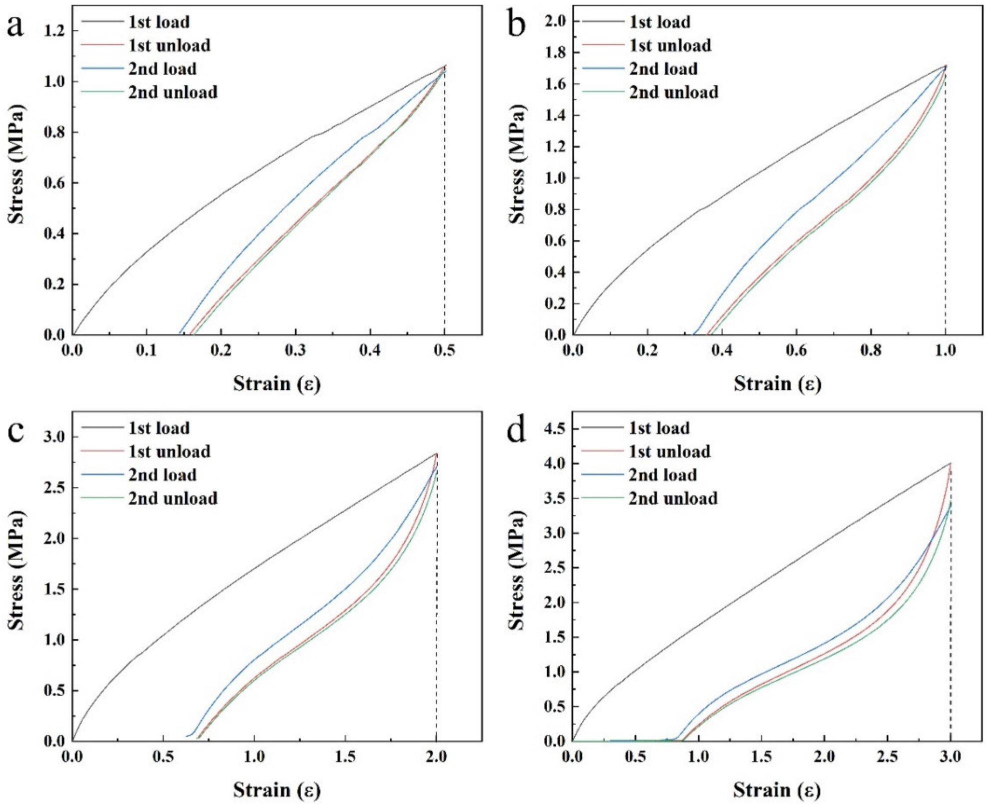

Figure S4. Stress-strain response of SiCB-MNR2/BR subjected to cyclic uniaxial tension: (**a**) ε = 0.5, (**b**) ε = 1.0, (**c**) ε = 2.0, (**d**) ε = 3.0.

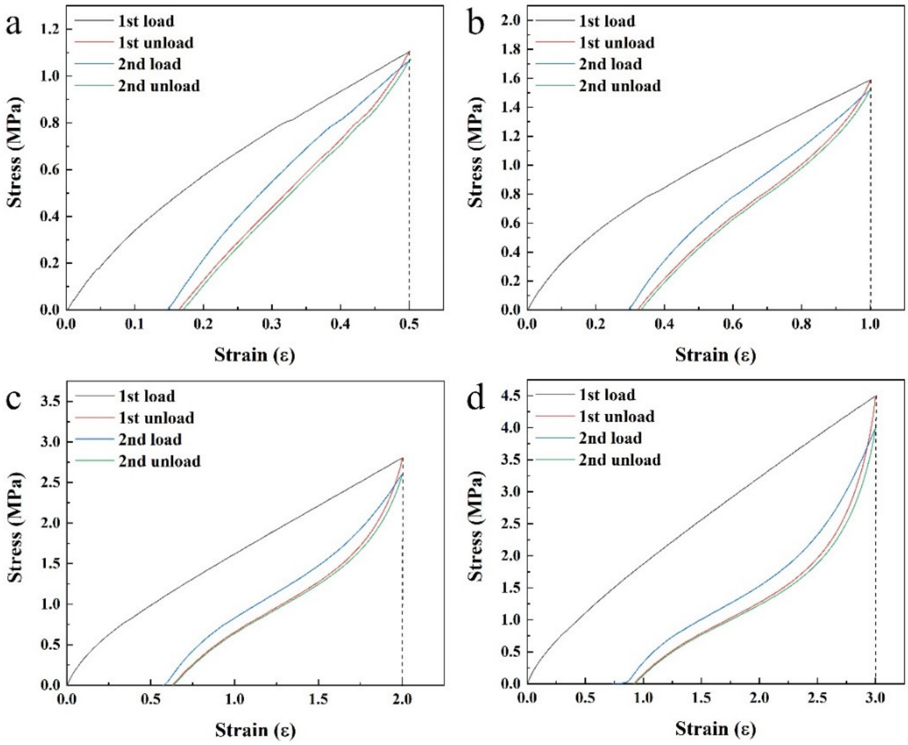

Figure S5. Stress-strain response of SiCB-MNR3/BR subjected to cyclic uniaxial tension: (**a**) ε = 0.5, (**b**) ε = 1.0, (**c**) ε = 2.0, (**d**) ε = 3.0.

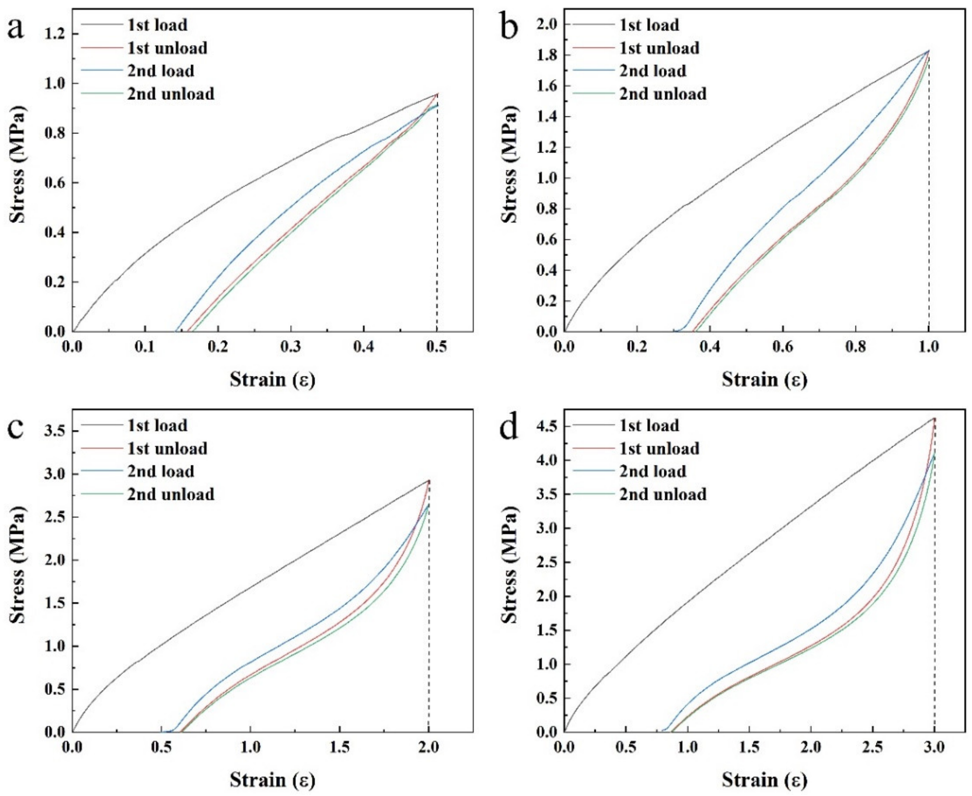

Figure S6. Stress-strain response of SiCB-MNR$_4$/BR subjected to cyclic uniaxial tension: (**a**) ε = 0.5, (**b**) ε = 1.0, (**c**) ε = 2.0, (**d**) ε = 3.0.

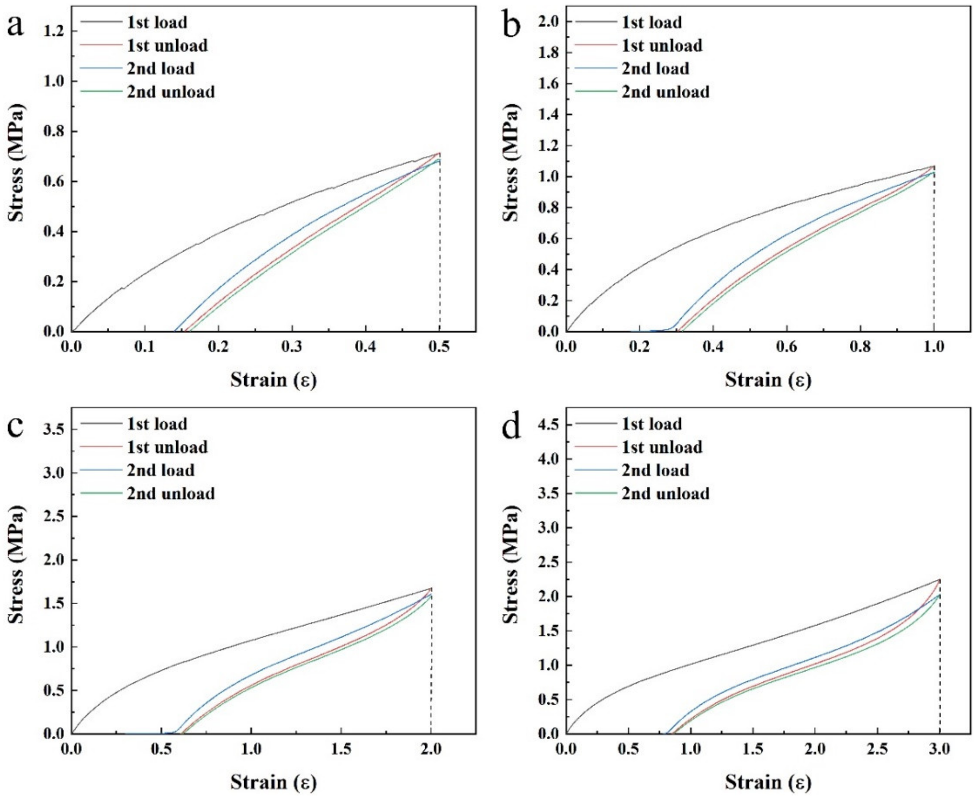

Figure S7. Stress-strain response of M$_1$SiCB-NR/BR subjected to cyclic uniaxial tension: (**a**) ε = 0.5, (**b**) ε = 1.0, (**c**) ε = 2.0, (**d**) ε = 3.0.

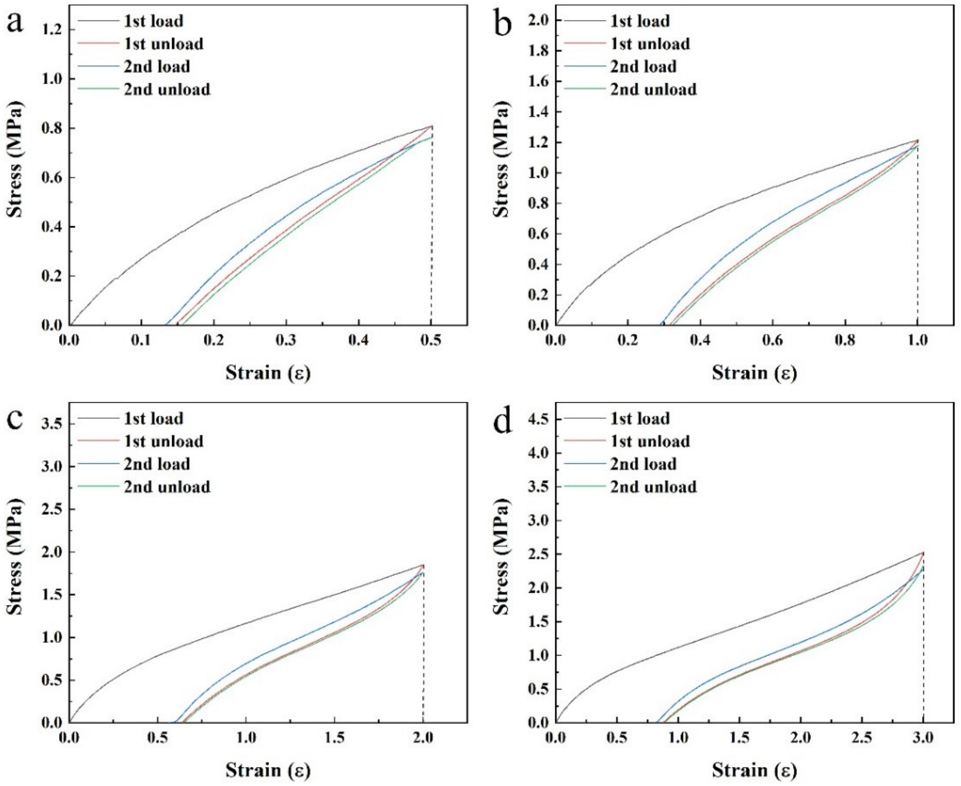

Figure S8. Stress-strain response of M_2SiCB-NR/BR subjected to cyclic uniaxial tension: (**a**) ε = 0.5, (**b**) ε = 1.0, (**c**) ε = 2.0, (**d**) ε = 3.0.

Reference

1. Gong, Wenjuan, and Rongrong Qi. "Graft Copolymerization of Maleic Anhydride onto Low-Molecular-Weight Polyisobutylene through Solvothermal Method." Journal of Applied Polymer Science 113, no. 3 (2009): 1520-28. https://dx.doi.org/10.1002/app.30020.
